# Neuroprotection by chitosan nanoparticles in oxidative stress-mediated injury

**DOI:** 10.1186/s13104-018-3162-7

**Published:** 2018-01-19

**Authors:** Bojun Chen, Jianming Li, Richard Ben Borgens

**Affiliations:** 1 Center for Paralysis Research, Department of Basic Medical Sciences, College of Veterinary Medicine, Purdue University, 408 S. University St., West Lafayette, IN 47907 USA; 2 University of Southern Indiana, 8600 University Blvd, Evansville, IN 47712 USA; 3 Weldon School of Biomedical Engineering, Purdue University, 206 S Martin Jischke Dr., West Lafayette, IN 47907 USA

**Keywords:** Chitosan, Nanoparticles, Oxidative stress, Neuroprotection

## Abstract

**Objective:**

Oxidative stress is a critical component of nervous system secondary injury. Oxidative stress produces toxic chemical byproducts including reactive aldehydes that traverse intact membranes and attack neighboring healthy cells. This secondary damage often leads to further patho-biochemical cascades that exacerbate the original insult. In this work, we investigate the therapeutic effects of chitosan nanoparticles on cell cultures exposed to oxidative stress.

**Results:**

We found chitosan nanoparticles can rescue BV-2 glial cells from death, but only for cells undergoing necrosis. Necrosis occurred when cultures were challenged with high concentrations of H_2_O_2_ (> 110 μM) whereas a slow and progressive loss of cultures was observed in more dilute (50–100 μM) peroxide applications. In the latter case, the primary mode of cell death was apoptosis. These studies revealed that while rescue of H_2_O_2_ challenged cultures was achieved for necrotic cell death, no such sparing was observed in apoptotic cells. Based on the current and cumulative data regarding the membrane fusogenic properties of chitosan, we conclude that chitosan neuroprotection arises from its membrane sealing effects. Consistent with this hypothesis is the observation that apoptotic cells did not exhibit early stage membrane damage. These in vitro results elucidate mechanisms by which membrane fusogens may provide therapeutic benefit.

**Electronic supplementary material:**

The online version of this article (10.1186/s13104-018-3162-7) contains supplementary material, which is available to authorized users.

## Introduction

Oxidative stress caused by reactive oxygen species (ROS) plays a key role in several neurodegenerative diseases as well as secondary injury in the central nervous system. ROS are highly toxic and can damage many biological molecules, including lipids, proteins, and/or nucleic acids. ROS can react with cell membrane lipids, leading to the initiation of lipid peroxidation (LPO) and increased membrane permeability [1, 2]. LPO can in turn, generate additional toxic species such as aldehydes (4-hydroxynonenal and acrolein).

The un-regulated generation of H_2_O_2_ is a well-known source of oxidative stress. H_2_O_2_ is the intermediate product in the conversion of O_2_^·−^ into H_2_O in the electron transport chain during mitochondria oxidative phosphorylation. Disruption of this equilibrium via cell injury can cause activated oxygen byproducts (O_2_^·−^ and H_2_O_2_) and overwhelm endogenous antioxidants such as superoxide dismutase, catalase, glutathione peroxidase, vitamin E and glutathione [3].

We previously showed chitosan based nanoparticles synthesized with and without a drug rescued PC-12 cells in an acrolein cell death model [4, 5]. The putative mode of cell preservation by chitosan was restoration of cell membrane integrity. Recovery of conduction was also demonstrated with chitosan in guinea pigs subjected to spinal crush [5]. In this work, we further investigate the neuroprotective properties of chitosan nanoparticles on BV-2 rat microglia cells challenged by H_2_O_2_. Similar to prior acrolein studies, this ROS injury model aims to mimic the biochemical mechanisms associated with CNS secondary injury.

## Main text

### Methods

#### Chi-DSNP preparation

The procedures and analysis of chitosan nanoparticles have been detailed previously [5]. Briefly, ionic gelation between chitosan polymer (200 kDa) and dextran sulfate polymer (DS) or sodium tripolyphosphate (TPP) polyanion was used. Two types of chitosan nanoparticles (chitosan-DS nanoparticles (~ 10 kDa) and chitosan-TPP nanoparticles) were synthesized. For technical reasons chitosan-DS nanoparticles (Chi-DSNPs) were employed in this study. Briefly, 0.1% chitosan was dissolved in 1% acetic acid and mixed for 12–18 h. 0.1% DS was prepared in DI water and filtered through 0.45 μm syringe filters. The DS solution was added drop-wise to the chitosan solution with continuous stirring for 1 h. The volume ratios for Chi-DSNPs were as follows: 5:3, 5:5, 5:8.5. During the DS-chitosan formation, the solution clouded when the volume ratio was above 5:3, indicating presence of nanoparticles. Following synthesis, the Chi-DSNPs were purified in 300 kDa dialysis tubing placed in DI water with stirring. The nanoparticle solutions were kept in 4 °C before use.

#### TEM

The morphology of ChiNPs were imaged via negative staining TEM. Briefly, one drop of Chi-NP solution was placed on a carbon grid and allowed to settle for 2 min. The grid was swished through a 2% uranyl acetate stain and the excess liquid removed. Samples were mounted and imaged using a Phillips CM-100 TEM operated at 100 kV with a 200 μm condenser aperture and 70 μm objective aperture.

#### Chi-DSNPS on BV-2 proliferation and viability

BV-2 mouse microglia obtained via Dr. Jau-Shyong Hong and Mrs. Belinda C. Wilson of NIH neuropharmacology group were maintained in DMEM supplemented with 0.044 M sodium bicarbonate, 10% fetal bovine serum and 100 U/ml penicillin and 100 μg/ml streptomycin. The cells were cultured in a 5% CO_2_ and 95% O_2_ incubator at 37 °C. 0.25 × 10^5^ cells using a 75 cm^2^ flask. For proliferation measurements in response to Chi-DSNPs, BV-2 cells were seeded at a density of 1 × 10^4^ cells/well in a 96-well plate. After overnight incubation, the cell medium was replaced with diluted NP solutions at a concentration of 0, 0.1, 0.2, 0.5 mg/ml, at a volume of 100 μl. For H_2_O_2_ challenge, the cell medium was replaced with H_2_O_2_ at 0, 50, 100, 200, and 300 μM for 20 h. In these experiments, cell proliferation was measured by using a WST-assay (Abcam) per manufacturer’s protocol and wells read with a plate reader at 450 nm. Four experiments were conducted in quadruplicate.

To measure BV-2 post-peroxide exposure viability, cells were seeded at 1 × 10^5^/well in a 12-well plate. After overnight incubation, the medium was replaced with a buffered H_2_O_2_ solution at 0 (control), 50, 100, and 5500 μM. Cells were imaged using an environmentally controlled Olympus IX81 microscope and proliferation tracked every 30 min for 20 h.

#### Mode of cell death

BV-2 cells were seeded at 1 × 10^5^/well in 12 well plates. Cells were exposed to H_2_O_2_ at 0 (control), 50, 100, and 5500 μM to induce cell death. The adherent cells were trypsinized gently and washed with phosphate buffer solution. Cells were incubated with 5 μl annexin V-FITC (Abcam) and 5 μl (50 mg/ml) of propidium iodide (Sigma) for 5 min at 25 °C. Cells were centrifuged gently and re-suspend in 200 μl binding buffer. 100 μl cell suspensions were placed on a glass slide, coverslip mounted and imaged using an Olympus IX81 at incubation times of 0 and 4 h for H_2_O_2_ (5500 μM) and at time 0 and 20 h for H_2_O_2_ (0, 50, 100 μM) with a dual filter set for FITC/PI.

#### Chi-DSNPs and neuroprotection

To assess the neuroprotective effects Chi-DSNPs, a concentration of 5500 μM H_2_O_2_ was chosen to induce BV-2 cell death. Here, BV-2 cells were seeded at a density of 0.5 × 10^5^ cells/well in a 24-well plate and cultured overnight. Afterwards, 10 μl of Chi-DSNPs at 1 mg/ml was administered at 0 and 15 min after H_2_O_2_ addition. Cell viability was measured by Trypan blue. BV-2 cells were also pre-incubated with 0.2 mg/ml Chi-DSNPs (pre-filtered with 1.2 or 5 μm syringe filter) for 4 h and subsequently exposed to 50 μM H_2_O_2_ for an additional 20 h. No treatment controls were BV-2 cells not exposed to H_2_O_2_, whereas injured controls were cells exposed to H_2_O_2_ but no nanoparticles were applied. Following 20 h culture, the WST-1 assay was used to quantify cell proliferation.

All data were represented as mean ± standard deviation. Statistical analysis was conducted using one-way ANOVA and a Tukey–Kramer post hoc test. A P value ≤ 0.05 was considered statistically significant.

### Results

#### Chi-DSNP characteristics

The bare Chi-NPs appeared as dark clustered spheres during TEM processing (Additional file 1: Figure S1). Larger globular CNPs were about 100 nm in diameter, while the majority of the clusters were 50 nm or smaller. Storage condition tests showed that this globular shape was maintained even after 2 weeks of air drying (data not shown). Dose–response characteristics of BV-2 cells exposed to Chi-DSNPs was assessed with WST-1. Results showed cell proliferation within 20 h (normalized to 0 mg/ml) was not affected significantly by Chi-DSNPS up to 0.5 mg/ml (P > 0.05, Additional file 1: Figure S1).

#### Time and dose dependent inhibition of cell proliferation induced by H_2_O_2_

**Fig. 1:**
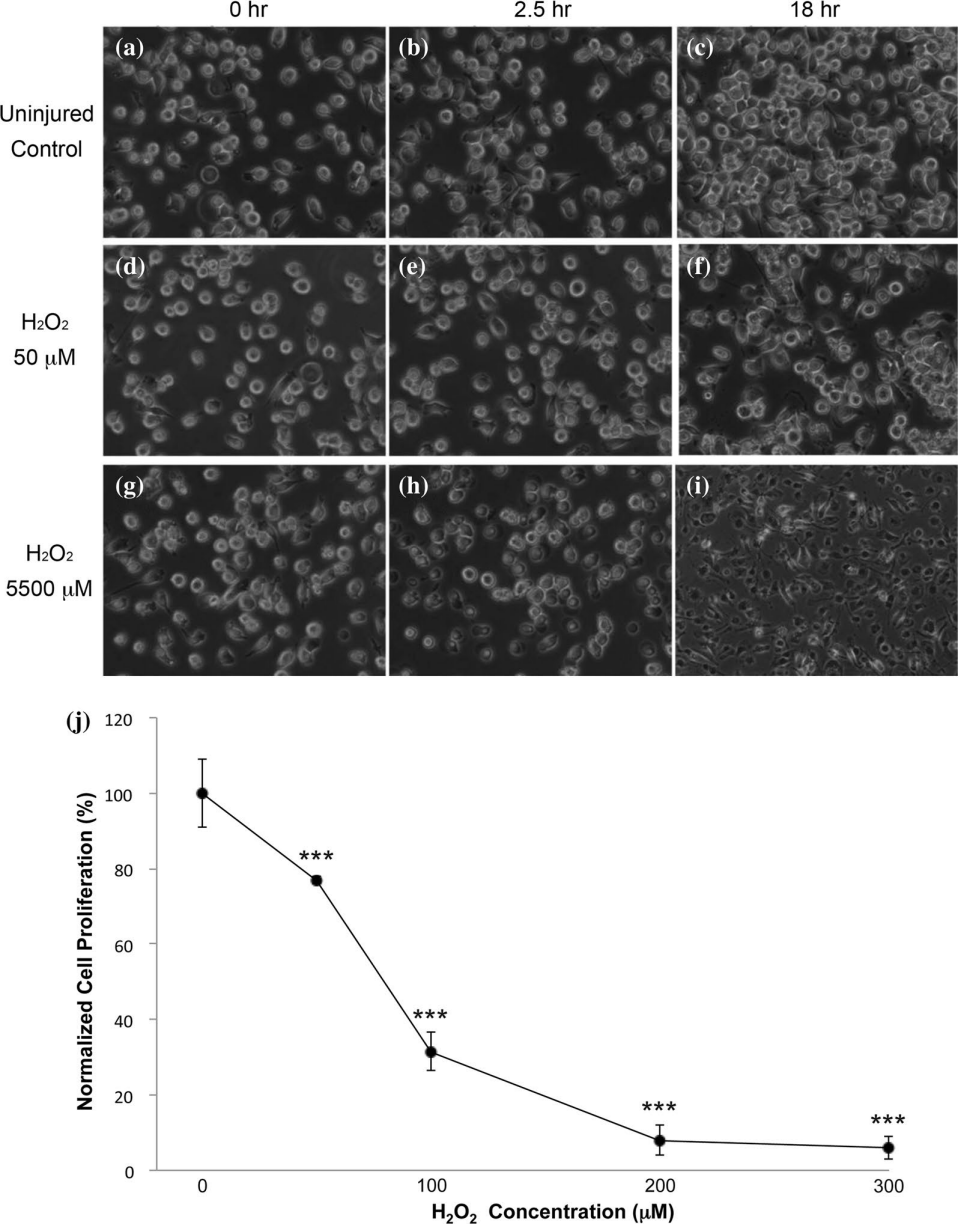
Morphological changes to BV-2 cells induced by H_2_O_2_. Phase contrast images of BV-2 cells incubated with different H_2_O_2_ concentrations (0, 50, and 5500 μM) at 0, 2.5 and 18, and 20 h. **a**–**c** Medium control. **d**–**f**, **j** H_2_O_2_ 50 μM. **g**–**i** H_2_O_2_ 5500 μM. **j** At ×40 high magnification, irregular cell surface and globular extrusions were observed which was highlighted by a light halo. Healthy cells appeared as spheres with white halos (**a**–**e**, **g**). Dead cells appeared as dark objects (**h**, **i**). H_2_O_2_ induced the inhibition of cell proliferation in all studied concentrations (***P < 0.001) after 20 h of exposure time. Decrease in cell proliferation was dose-dependent (P < 0.001). All H_2_O_2_ treated groups were normalized and compared with the control group. Data is represented as mean ± SD

H_2_O_2_ reduced BV-2 cell proliferation within the initial 20 h when assessed with WST-1 (Fig. 1). At H_2_O_2_ concentration > 200 μM, no difference was detected with WST assay as death saturated the populations. Cells at H_2_O_2_ challenge time points of 0, 2.5 h, and 18 h were selected to evaluate changes to cell morphology. In the uninjured (0 μM H_2_O_2_) control groups, (Fig. 1A–C), cells appeared adherent with elongated processes and normal proliferation. In 50 μM H_2_O_2_ group (D–F), cell morphology was distorted after only 4 h. In (F), multiple dead cells formed clumps and altogether appeared as non-viable cultures. The surface of these cells was very irregular with globular inclusions. In the 5500 μM hydrogen peroxide group (G–I), cells started retracting their processes after only 1 h incubation. Cell blebs were formed and the swelling of the cytoplasm was observed (data not shown). These cells later darkened, indicating near or actual cell death. Multiple small bright spots were detected in single dead cells, suggesting breaches in the cell membranes (I).

**Fig. 2:**
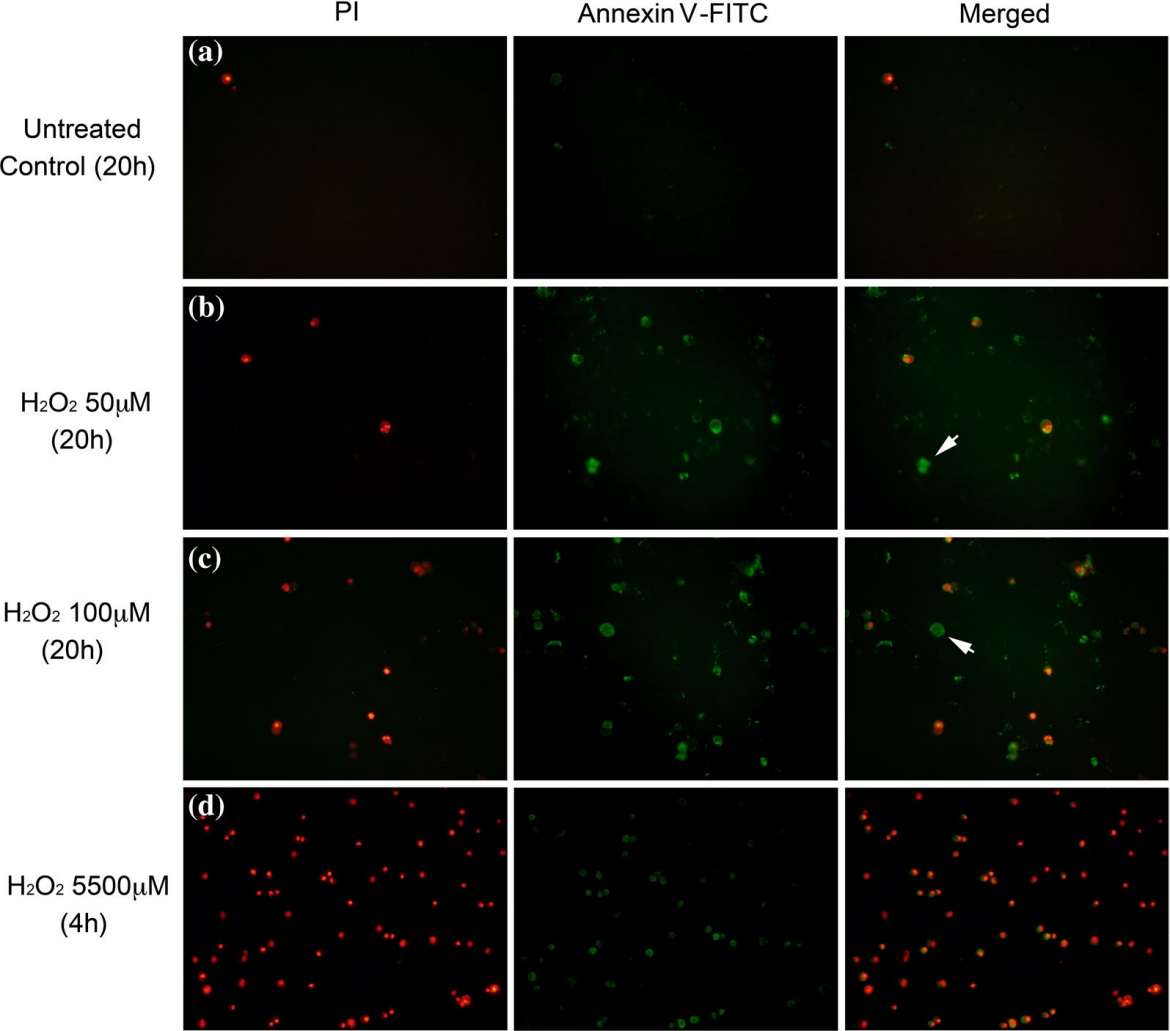
Fluorescence images of BV-2 cells stained with PI and FITC-annexin V. BV-2 cells challenged with H_2_O_2_ at: Row **a**: medium control—20 h, Row **b**: 50 μM—20 h, Row **c**: 100 μM—20 h, and Row **d**: 5500 μM—4 h. After H_2_O_2_ treatments, cells were collected, washed and stained with both PI and FITC-annexin V fluorophores. PI and FITC signals were captured separately by a fluorescence microscope and the images were merged to identify the localization of the stains. PI and FITC signal was represented as red and green color, respectively. White arrows highlight cells undergoing apoptosis

Mode of cell death was determined PI and annexin-FITC stains. Photomicrographs Fig. 2d–f show BV-2 cells treated with 5500 μM H_2_O_2_ possessed bright red (PI) nuclei and small evidence of FITC on their membranes vs control. We did not detect any cells stained only with FITC. Interestingly, the treatment of 50 μM (b) and 100 μM (c) H_2_O_2_ resulted in some staining with annexin-V/PI, but also led to cells stained only with FITC. This suggests that a long exposure to a low H_2_O_2_ dose induced mostly apoptotic cell death, with only minor necrosis.

#### Chi-DSNPs on BV-2 viability after H_2_O_2_ exposure

**Fig. 3:**
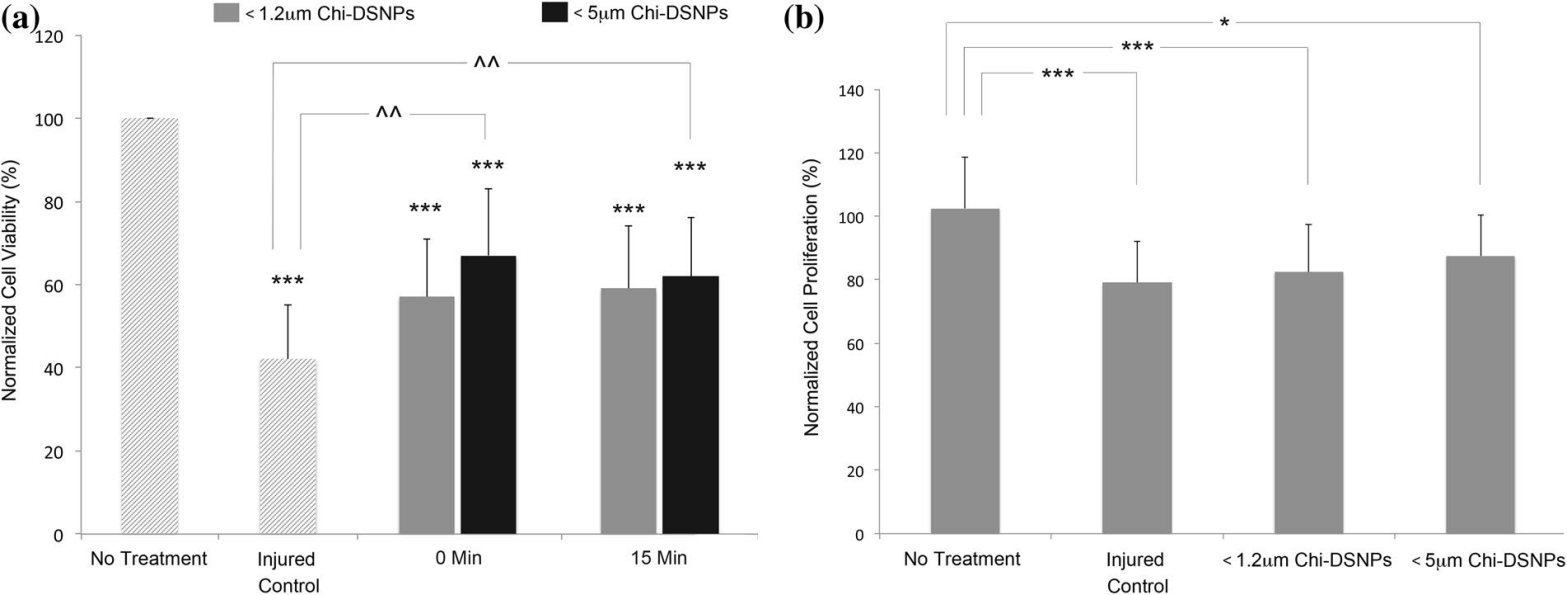
Neuroprotection by Chi-DSNPs on H_2_O_2_ Challenged BV-2 Cells. **a** BV-2 cells were incubated with 0.1 mg/ml Chi-DSNPs of different sizes immediately or 15 min after challenging with 5500 μM H_2_O_2_. The data in H_2_O_2_ treated groups was normalized and compared with medium–medium treated group (***P < 0.001). The administration of 5 μm filtered group Chi-DSNPs, both at 0 min and post 15 min, enhanced cell viability significantly compared with H_2_O_2_-medium treated group (^^^^P < 0.01). Trypan blue was used to detect dead cells. All data was represented mean ± SD. **b** BV-2 cells were pre-incubated with 0.2 mg/ml Chi-DSNPs (pre-filtered with 1.2 or 5 μm syringe filter) for 4 h and then challenged with 50 μM H_2_O_2_ for 20 h. Cell proliferation was detected by WST assay. Data represented as mean ± SD. Cell proliferation was inhibited significantly by long-exposure of H_2_O_2_. An increasing of cell proliferation was observed in H_2_O_2_ challenged cells treated with 1.2 μm filtered Chi-DSNPs group (1.2 μm-NPs), and no difference was detected between injured control and Chi-DSNPs treatment (*P < 0.05, **P < 0.01, ***P < 0.001)

BV-2 cell viability after the administration of Chi-DSNPs at 0 and 15 min after exposure to 5500 μM H_2_O_2_ was measured by Trypan blue. Results (Fig. 3) showed at a 2.5 h exposure time, 5500 μM H_2_O_2_ induced almost 60% cell death vs untreated control groups (P < 0.001) whereas 5 μm-filtered Chi-DSNPs preserved cells by 20% (P < 0.01). No difference in the timing of CHI-DSNPs (0 or 15 min) application was detected. Additionally, a 30 min delayed administration of Chi-DSNPs did not provide further beneficial effect (data not shown). At low dose, WST-1 was used to determine the protective effects of Chi-DSNPs on H_2_O_2_ challenge (Fig. 3b). Findings show a 20% decrease in cell proliferation after a 20 h administration of 50 μM H_2_O_2_ (P > 0.05). Pre-treating the cells with either 1.2 or 5 μm filtered Chi-DSNPs at 0.2 mg/ml for 4 h prior to H_2_O_2_ challenge did not statistically improve cell proliferation.

### Discussion

Chitosan is a commonly used polymer in biomaterials research due to its good biodegradability, biocompatibility and accessibility for surface modification [6, 7]. Previously, we showed chitosan nanoparticles exhibited neuroprotective effects in an acrolein-challenged PC-12 cell model [7]. In this work, we extend the line of investigation to BV-2 cells, an immortalized rat microglial line that emulates the characteristics of primary microglia, displaying similar inflammatory response and phagocytic capacity [8]. H_2_O_2_ was used as the challenge molecule since it is a common oxidative stressor for modeling many neurodegenerative diseases. For instance, Liu et al. [9] reported a significant elevation of intracellular level of H_2_O_2_ 30 min after a weight drop induced SCI. These high levels of H_2_O_2_ were maintained for over 11 h. H_2_O_2_ was suggested to arise from O_2_^·−^. The post-injury activation and sustained increase of H_2_O_2_ indicate that it was not just an immediate response to SCI, and implicate H_2_O_2_ in secondary injury processes associated with SCI [3].

To mimic the acute release of H_2_O_2_ post SCI injury, we first constructed the H_2_O_2_ toxicity profile with BV-2 cells at exposure ranges estimated in vivo or in other experimental preparations [10–12]. The experimental results show short term exposure to H_2_O_2_ (< 50 μM) suppressed cell proliferation. Further assessment via morphological analysis and with annexin-V/PI staining (reviewed in [3]) highlight differences in mode of cell death. Acute exposure to high peroxide levels (5500 μM) induced cell membrane damage and rapid necrosis in less than 2.5 h whereas low levels of peroxide caused mostly apoptosis, which was evident in the annexin V staining. This data is not unexpected, and similar to other instances of ROS-SCI induced cell death, which is usually a combination of necrosis and apoptosis [3, 13–15]. Administration of chitosan nanoparticles showed chitosan nanoparticles protected microglia cells challenged with 5500 μM hydrogen peroxide (Fig. 3) for 15 min. However, no improvement in cell proliferation was observed between control and the chitosan nanoparticle group was found when BV-2 cells were pre-treated with nanoparticles and subsequently exposed to 50 μM hydrogen peroxide for 20 h. The chitosan nanoparticles themselves were well tolerated by the BV-2 cells based on proliferation assays.

The putative neuroprotective mechanism for chitosan is sealing of damaged cell membranes in a manner similar to fusogens such as polyethylene glycol [6, 16]. Therefore, it is consistent to expect necrotic cells to be sensitive to chitosan rescue. In contrast, low dosages of peroxide (50 μM) caused cellular apoptosis—a process that is biochemically driven, primarily irreversible and does not involve membrane damage at the onset. This was confirmed both morphologically and via annexin-V staining. For apoptotic cells, chitosan NPs had no therapeutic impact. While some studies also report anti-oxidative properties of chitosan, especially if pre-incubated with cells, such investigations are impractical outside of in vitro cultures and may not reveal useful insights into disease treatment [17, 18]. Again, our current findings also suggest pre-treatment with chitosan NPs does not have a meaningful effect on low-dose H_2_O_2_ challenge. Thus, we conclude that neuroprotection by chitosan nanoparticles is largely due to a physical sealing of cell membrane breaches, an observation that has been corroborated by our prior work in chitosan, poloxamers, poloxamines, and PEG [3, 4, 6, 19–24]. Due to the versatility of chitosan nanoparticles as potential drug delivery reservoirs/vehicles, these preliminary results offer support for further therapeutic investigations.

## Limitations

This work was conducted with cell cultures and it is unknown if the results are applicable in vivo or if there may be longer-term benefits from nanoparticle administration.

### Additional file

**Additional file 1.** Chitosan nanoparticles. Transmission electron micrographs of chitosan nanoparticles. Most particles were 100 nm or less in diameter. Corresponding table shows Chi-DSNPs did not significantly inhibit cell proliferation after 20 h incubation at different concentrations (0, 0.1, 0.2, 0.5 mg/ml).

## Abbreviations

ROS: reactive oxygen species
LPO: lipid peroxidation
NPs: nanoparticles
DS: dextran sulfate
TPP: tripolyphosphate
Chi-DSNPs: chitosan-DS nanoparticles
PS: phosphatidylserine
PI: propidium iodide
