# Supplementary figures and images for: Neuroprotection by chitosan nanoparticles in oxidative stress-mediated injury
Source: BMC Res Notes. 2018 Jan 19;11:49. doi: 10.1186/s13104-018-3162-7 (PMC5775548; doi:10.1186/s13104-018-3162-7)

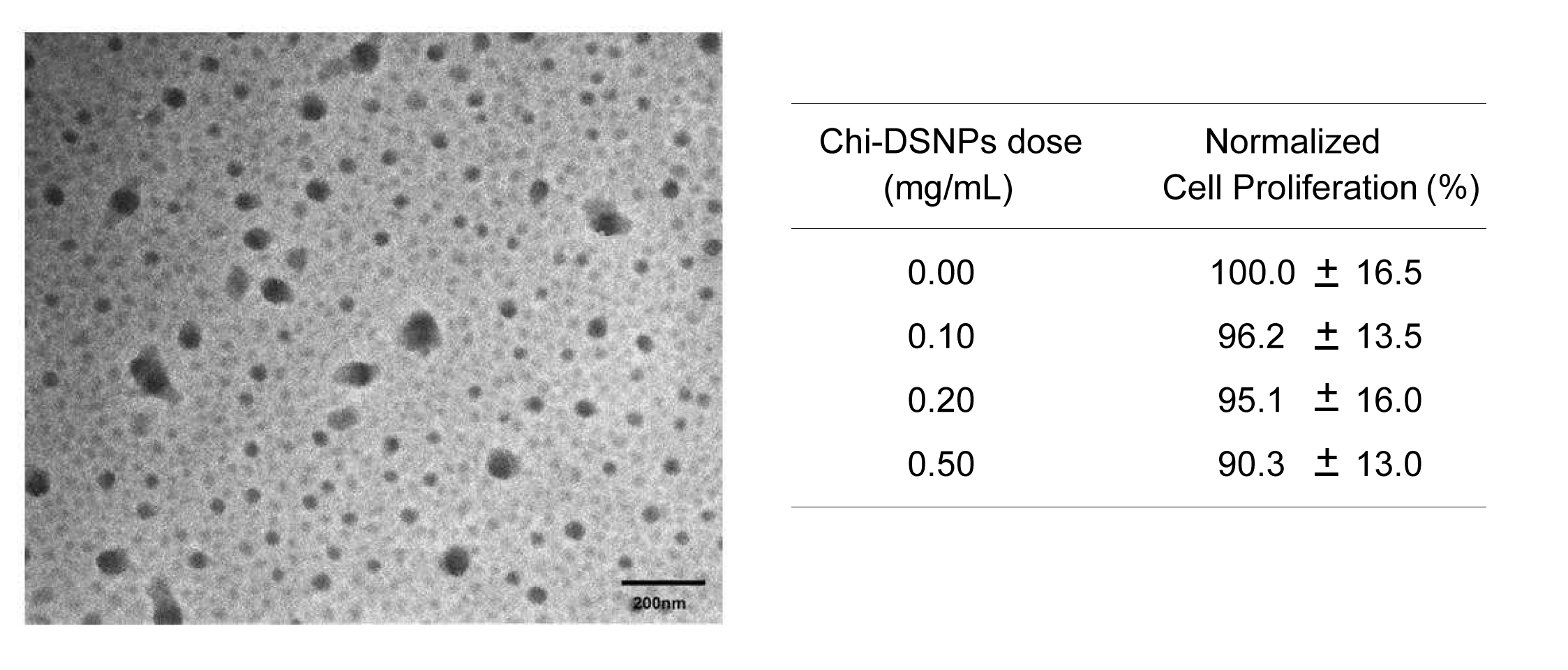

Supplement: Supplementary file 1 — Additional file 1. Chitosan nanoparticles. Transmission electron micrographs of chitosan nanoparticles. Most particles were 100 nm or less in diameter. Corresponding table shows Chi-DSNPs did not significantly inhibit cell proliferation after 20 h incubation at different concentrations (0, 0.1, 0.2, 0.5 mg/ml). [file 13104_2018_3162_MOESM1_ESM.jpg]
